# Non-functioning pituitary microadenoma in children and adolescents: Is follow-up with diagnostic imaging necessary?

**DOI:** 10.1007/s12020-022-03212-7

**Published:** 2022-10-17

**Authors:** Camilla Borghammar, Ashkan Tamaddon, Eva-Marie Erfurth, Pia C. Sundgren, Peter Siesjö, Maria Elfving, Margareta Nilsson

**Affiliations:** 1 Skåne University Hospital, Department of Clinical Sciences, Pediatrics, Pediatric Endocrinology, Lund University, Lund, Sweden; 2 Skåne University Hospital, Department of Clinical Sciences, Medical Imaging and Physiology, Lund University, Lund, Sweden; 3 Skåne University Hospital, Department of Clinical Sciences, Endocrinology, Lund University, Lund, Sweden; 4 Department of Clinical Sciences, Radiology, Lund University, Lund, Sweden; 5 Lund Bioimaging Center, Lund University, Lund, Sweden; 6 Skåne University Hospital, Department of Clinical Sciences, Neurosurgery, Lund University, Lund, Sweden

**Keywords:** Pituitary microadenoma, Pituitary cyst, Children, MRI, Follow-up

## Abstract

**Purpose:**

No consensus exists regarding follow-up recommendations for suspected pituitary microadenoma in children. To address this knowledge gap, we investigated the growth potential of pituitary solid and cystic lesions <10 mm in children and evaluated the accuracy of magnetic resonance imaging (MRI) measurements.

**Methods:**

The children included were <18 years at first pituitary MRI and radiologically diagnosed with a non-functioning microadenoma or cyst <10 mm. Lesion size at first and latest MRI as well as all individual MRI examinations were re-evaluated.

**Results:**

In total, 74 children, median age 12 years (range 3–17), had a non-functioning microadenoma, probable microadenoma, or cyst. Of these, 55 underwent repeated MRI (median 3, range 2–7) with a median follow-up of 37 months (range 4–189). None of the pituitary lesions without hormonal disturbances increased significantly during follow-up. Two radiologists agreed that no lesion could be identified in 38/269 (14%) MRI examinations, and in 51/231 (22%) they disagreed about lesion location. In 34/460 (7%) MRI measurements size differed >2 mm, which had been considered significant progression.

**Conclusion:**

Non-functioning pituitary microadenoma in children has small size variations, often below the spatial resolution of the scanners. We suggest lesions <4 mm only for clinical follow-up, lesions 4–6 mm for MRI after 2 years and ≥7 mm MRI after 1 and 3 years, with clinical follow-up in between. If no progression, further MRI should only be performed after new clinical symptoms or hormonal disturbances.

## Introduction

Pituitary adenomas are usually benign lesions with a lower incidence in children and adolescents than in adults [1, 2]. In the entire population, pituitary adenomas account for 2.6–8.5% of all pituitary tumours [3]. In children, pituitary tumours account for less than 3% of all intracranial tumours [1, 3]. However, a recent study found that in children below the age of 19 pituitary tumours made up 19.7% of all brain tumours and 70.9% of these were adenomas [4].

In studies performed on volunteers and in unselected patients, the estimated prevalence of pituitary incidentalomas (i.e., accidental findings with no symptoms correlated to the hypothalamic-pituitary area) is as high as 20–22.5% [1, 5]. In autopsy studies, pituitary incidentalomas are present in 14.4–25% of the population. In children, the prevalence of incidentalomas is not so well described, but a few autopsy studies have shown a low prevalence of about 1.5% [1, 2, 5].

Both microadenomas (diameter < 10 mm) and macroadenomas (≥10 mm) are found in children, but microadenomas are much more frequent and often found incidentally [6]. Pituitary adenomas in children are mainly sporadic, but a few are part of a syndrome (e.g., MEN-1 or familial mutation of the AIP gene) [1–3, 7], and some are hormone producing. In children, prolactinoma is the most common hormone-producing adenoma, followed by ACTH-producing and GH-producing adenoma [7, 8].

When pituitary adenoma needs treatment, the choice of treatment depends mainly on the size of the lesion and hormone status. Intervention may consist of medical treatment, surgery, and/or radiation [1, 2]. For non-functioning microadenoma or cystic pituitary lesions <10 mm, patients often enter a follow-up program with clinical assessment and repeated magnetic resonance imaging (MRI) to monitor possible growth potential both in the short and long term.

Increased use of MRI and improved technique provides the opportunity to detect small pituitary lesions and during recent years, it has become a more common finding. Focal findings in the pituitary gland are usually adenomas and cystic lesions. Cysts are divided into Rathke’s cyst, usually situated in the midline, and off-midline cysts, which might represent a cystic microadenoma. As these lesions are often close to the resolution of MR scanners, considerable diagnostic overlap is likely.

MRI protocol requires both sagittal and coronal planes in thin sections (2 or 3 mm) and small field of view focused on the pituitary gland. T1-weighted sequences before and after intravenous gadolinium contrast are crucial for evaluation of a solid microadenoma, visualized as an area with delayed contrast enhancement. A cystic lesion is depicted as a liquid-filled tumour on T2-weighted sequences or a lesion with lack of contrast enhancement on T1-weighted gadolinium sequences. Sometimes dynamic post-contrast MRI sequences highlight a small microadenoma as an area with delayed contrast enhancement, only visible during the first arterial phase of the contrast injection. Most often a pituitary adenoma is adequately demonstrated on standard acquisition after contrast administration [9].

We have limited knowledge about the natural history of a microadenoma, including whether it presents as a microadenoma and later develops into a macroadenoma and, if so, whether its size increases over a shorter or longer period. A few studies, performed mainly in adults, found that 3–13% of incidentalomas increased in size between 18 months and 8 years [10–13]. The increase in size after 18 months, however, was very small, only 1–2 mm [13], a change in size that is close to MRI resolution. In a few studies of children with small non-functioning pituitary lesions and repeated MRI, only 1 of 145 children had a pituitary lesion that increased in size 4 mm after 6 years, but it still remained < 10 mm [6, 14–16]. Nevertheless, there is a lack of consensus on recommendations of the optimal clinical and radiological follow-up of a microadenoma in children.

The first aim of this study was to investigate the growth potential of pituitary microadenomas and cystic pituitary lesions less than 10 mm in children examined with repeated MRI scans and to evaluate presenting symptoms, hormonal status, and clinical status at the time of the first MRI. The second aim was to evaluate how accurate and reproducible detection and measurements of the pituitary microadenomas and cysts were between two different reviewers on individual MRI scans.

## Patients and methods

### Patient inclusion

**Fig. 1:**
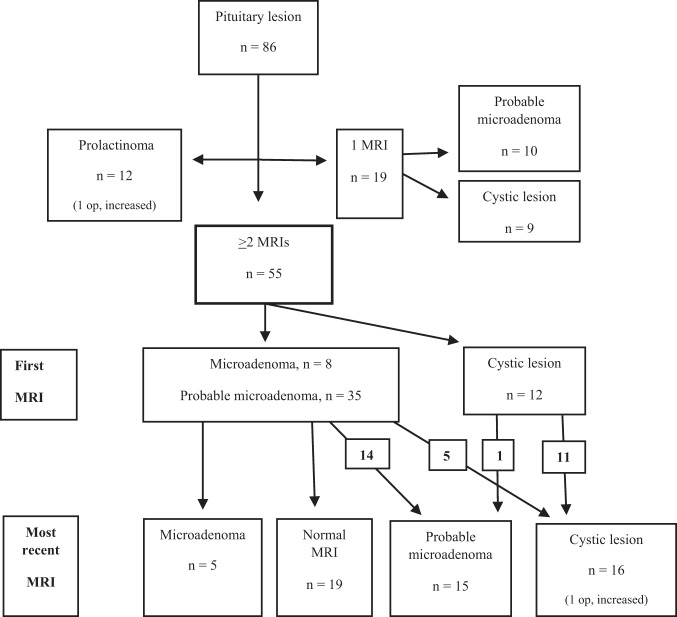
Summary of the original MRI reports of children with pituitary microadenoma, probable microadenoma, cystic lesion, and prolactinoma. Op Operation, MRI Magnetic resonance imaging

This retrospective study included children younger than 18 years old referred for a dedicated MRI scan of the pituitary gland between 2007 and 2017 at Skåne University Hospital, Sweden. The initial patient cohort consisted of 696 patients identified through a search in Picture Archiving and Communication System (PACS). MRI statements and actual age at first examination were checked, and 614 patients were excluded for the following reasons: ≥18 years old, craniopharyngioma diagnosis, lack of pituitary pathology, macroadenoma, microadenoma-producing ACTH, GH or TSH, or incomplete examination. Children with a prolactinoma were included, as we also wanted to explore the methodical accuracy of visualizing the size of lesions, and all of these had repeated MRI scans. As a result of this registry search 82 patients met the inclusion criteria – i.e., <18 years old at first MRI, radiological diagnosis of a non-functioning pituitary microadenoma or cyst <10 mm, or prolactinoma. An additional 11 patients were included from a search in the medical diagnosis registry – ICD D352 (benign pituitary tumour) and D443 (tumour with unknown/uncertain nature in the pituitary gland) – in BORISS (Paediatric Oncology Register in the South Region of Sweden) and in medical records. Seven of the initial 93 patients were excluded after review due to pituitary lesion being ≥10 mm, a diagnosis other than the included ones, or no medical records available. In the end, 86 patients were included in the study, which included 12 patients with a prolactinoma (Fig. 1).

### Clinical evaluation and management

We evaluated the clinical management of pituitary lesions based on the diagnosis suggested by the first MRI report: microadenoma, probable microadenoma, cystic lesion (including Rathke’s cleft cyst and off-midline cyst), and prolactinoma (pituitary lesion and elevated prolactin). We used the term probable microadenoma when the original report revealed some uncertainty in the finding, but a follow-up was planned. The suggested diagnosis of the lesion at the initial and final scan was noted.

For each patient, the following clinical data were collected from the medical records: presenting symptoms, first MRI scan, and probable diagnosis, treatment, and planned follow-up, results of ophthalmological examination, surgery, and histopathological examination, and data regarding sex, age, weight, and height. Serum hormone levels were also noted (TSH, fT4, IGF-1, FSH, LH, estradiol, testosterone, SHBG, prolactin, ACTH, cortisol, and 24 h urine cortisol x 2) at the initial MRI.

### MRI imaging review for lesion size

The size of the lesion at the first and most recent MRI was reviewed by an experienced neuroradiologist since the lesion’s size was not always well described in the original MRI report. When patients had multiple MRI examinations, a change in size of >2 mm was considered a progression or reduction of lesion size.

### MRI re-evaluations – measurement accuracy

All individual MRI examinations were independently re-evaluated by two neuroradiologists – a senior neuroradiology resident with more than 7 years of radiological experience and a neuroradiologist with more than 20 years of experience. The MRI scans were randomly assigned with a unique number and all examinations were evaluated in a random order. Both reviewers were blinded to all clinical information, including the time of examination and whether other examinations existed for the same patient. The pituitary gland was divided into quarters in the coronal and in the sagittal plane. Each reviewer was asked to report if they could identify the lesion within the pituitary gland, and, if a lesion was seen, to note what quarter in the pituitary gland the lesion was located. In patients where both reviewers indicated the same location it was considered likely that the same lesion was observed. At last, for evaluation of inter-observer agreement the reviewers’ measurements (height, width, and depth) were compared. An estimated difference in size of >2 mm was noted – i.e., a difference in size in two consecutive scans was considered a progression. The same calculation was performed for a difference of >3 mm. The differences were compared for the subgroups microadenoma, probable microadenoma, cystic lesion, and prolactinoma. Technical data from the MRI such as field strength, sequence type, and slice thickness were noted together with the size and appearance of the tumour (signal characteristics). MRI examinations performed at 1.5 T and 3.0 T were included.

MRI parameters were somewhat heterogeneous but generally included a small field of view, T1- and T2-weighted sequences of the pituitary gland, with gadolinium-enhanced coronal and sagittal T1-weighted sequences, and slice thicknesses of 2 to 3 mm with little or no interslice gap. After these assessments were completed, the individual scans were again assigned to the individual patient in chronological order for statistical analysis of the diagnosis suggested in the primary report.

The study was approved by the Regional Ethics Committee, Medical Faculty, Lund University, Sweden (approval no. 2017/849)/ (Dnr 2017/849).

### Statistical analysis

Descriptive data were expressed as count, percentage, and median (minimum–maximum). For all MRI examinations, the largest dimension of the pituitary lesion was used for statistical analysis. For inter-observer agreement of the lesion size, kappa (95% CI) were calculated separately for microadenoma, probable microadenoma, cystic lesion, and prolactinoma (kappa < 0.2 poor, 0.21–0.40 fair, 0.41– 0.60 moderate, 0.61–0.80 good, and > 0.80 very good). All analyses were performed in Stata SE 16.0. Confidence intervals (CI) were set to 95%.

## Results

### Clinical evaluation

#### Microadenoma, probable microadenoma, and cystic lesion

At the first MRI, 74 children had a lesion and suggested radiological diagnosis, according to the primary report, of a pituitary microadenoma, probable microadenoma, or cystic lesion (Fig. 1). Their median age was 12 years, 43 (58%) were girls, and the majority (*n* = 48, 65%) were pubertal (Table 1). Presenting symptoms are demonstrated in Table 2. The most common symptom was precocious puberty (*n* = 27, 36%) and growth disturbances (*n* = 22, 30%). Of these 74 children, 32 had received hormonal treatment (Table 3).

**Table 1:** Demographics, number of MRI scans, and follow-up time in children with pituitary microadenoma, probable microadenoma (PA), cystic lesion (CL), and prolactinoma

	≥ 2 MRIs microadenoma, PA, CL *n* = 55	1 MRI PA, CL *n* = 19	Prolactinoma *n* = 12
Age at first MRI scan median (range), years	12 (4–17)	10 (3–17)	16 (12–17)
Females, *n* (%)	31 (56%)	12 (63%)	8 (67%)
Pubertal, *n* (%)	35 (64%)	13 (68%)	12 (100%)
Ophthalmological examination, *n* (%)	3 (5%)^a^	1 (5%)^a^	7 (58%)^a^
Operation, *n* (%)	1 (2%)	0	1 (8%)
Hormone treatment, *n* (%)	22 (40%)	10 (53%)	12 (100%)
Number of MRI scans, median (range)	3 (2–7)	N A	5.5 (2–11)
Follow-up time, median (range), months	37 (4–189)	N A	75.5 (26–136)

^a^Normal result of ophthalmological examination in all cases. *NA* Not applicable

**Table 2:** Presenting symptoms: pituitary microadenoma, probable microadenoma (PA), cystic lesion (CL), and prolactinoma

Presenting symptoms: pituitary microadenoma, PA, CL, and prolactinoma	≥ 2 MRIs micoradenoma, PA, CL *n* = 55	1 MRI PA, CL *n* = 19	Prolactinoma *n* = 12
Precocious puberty, *n* (%)	20 (36%)	7 (37%)	0
Growth disturbances, *n* (%)	13 (24%)	9 (47%)	1 (8%)
Delayed puberty, *n* (%)	6 (11%)	1 (5%)	1 (8%)
Headache, *n* (%)	5 (9%)	0	3 (25%)
Fatigue, *n* (%)	3 (5%)	0	0
Pubertal arrest, *n* (%)	3 (5%)	0	0
Pituitary insufficiency, *n* (%)	2 (4%)	0	0
Ptosis, *n* (%)	1 (2%)	0	0
Stereotype movements, *n* (%)	1 (2%)	0	0
Focal epilepsy, *n* (%)	1 (2%)	0	0
Hyperprolactinemia (risperidone treatment), *n* (%)	0	1 (5%)	0
Secondary amenorrhea, *n* (%)	0	1 (5%)	1 (8%)
Galactorrhoea, *n* (%)	0	0	5 (42%)
MEN 1 syndrome, *n* (%)	0	0	1 (8%)

**Table 3:** Hormone treatment: pituitary microadenoma, probable microadenoma (PA), cystic lesion (CL), and prolactinoma

Hormone treatment: pituitary microadenoma, PA, and CL	≥ 2 MRIs micoradenoma, PA, CL *n* = 55	1 MRI PA, CL *n* = 19	Prolactinoma *n* = 12
Growth hormone, *n* (%)	6 (11%)	4 (21%)	0
GnRH analogue, *n* (%)	5 (9%)	4 (21%)	0
Testosterone, *n* (%)	4 (7%)	2 (11%)	0
Thyroxine, *n* (%)	3 (5%)	0	0
Estrogen, *n* (%)	2 (4%)	0	0
Multiple pituitary hormone substitution, *n* (%)	2 (4%)	0	0
Dopamine agonist	0	0	12 (100%)

Repeated MRI was performed on 55 children, the main study population. The median number of repeated MRI in this group was 3 (2–7), with a median follow-up time of 37 months (4–189 months) (Table 1). One patient with two cystic lesions underwent surgery due to suspected pressure on the pituitary stalk. Two patients had multiple pituitary insufficiencies, one of them with congenital onset. Two patients had GH deficiency. Levels of sex hormones, gonadotropins, and IGF-1 that deviated from expected levels at a certain age were due to early or late puberty. For presenting symptoms and hormone treatment there were no significant differences between the groups diagnosed with a microadenoma/probable microadenoma/cystic lesion.

#### Prolactinoma

In the 12 children with prolactinoma, eight (67%) were females and their median age at the initial MRI was 16 years (Table 1). All patients were subsequently treated with dopamine agonists resulting in diminished lesion size, but one patient had surgery due to poor effect of pharmacological treatment. The median number of MRIs was 5.5 (2–11), and the median follow-up time was 75.5 months (26–136 months). The most common presenting symptom (Table 2) was galactorrhea (42%).

### Clinical management consistent with the primary MRI reports and from medical records

Among those 55 children with repeated MRI, 43 patients (Fig. 1) were diagnosed with a microadenoma or probable microadenoma at first MRI, 19 had no radiologically significant pituitary lesion at the most recent MRI, five were ultimately diagnosed with a microadenoma, 14 were diagnosed with probable microadenoma, and five were diagnosed with a cystic lesion. Three of the children ultimately diagnosed with microadenoma and five of the children ultimately diagnosed with probable microadenoma were scheduled for a new MRI after the data collection. Eight children had no planned MRI and underwent only clinical follow-up. Among those with probable microadenoma, one patient was lost to follow-up because of bad compliance and two patients were lost to follow-up because of unavailable recent medical records. In 12 patients, a cystic lesion was the initial diagnosis (Fig. 1). As the result of the most recent MRI, one of these patients was diagnosed as a probable microadenoma. An additional five patients from the microadenoma/probable microadenoma group were finally diagnosed with a cystic lesion. Of the 16 patients diagnosed with a cyst at the most recent MRI, five were planned to undergo further MRI after the closure of this study. Two underwent clinical follow-up and nine had no planned follow-up. One patient with a cystic lesion ultimately diagnosed as a probable microadenoma was lost to follow-up because of bad compliance.

One child in the main study population of 55 children had a cystic lesion that increased in size. The lesion was 4 mm at the initial MRI, and a small new cyst appeared during follow-up. Five years after the first MRI, the patient developed multiple hormone deficiency. A new MRI scan showed an enlargement for one of the two cysts, which now measured 6 mm. This patient underwent transphenoidal surgery, and the microscopic examination showed a benign cyst.

In total, 19 of 74 patients underwent only one MRI (Fig. 1). These 19 patients were re-evaluated using MRI and a multidisciplinary decision was made regarding diagnosis and follow-up: seven cases were considered normal, nine were diagnosed as a cystic lesion with no need for any further follow-up, and three were diagnosed with probable microadenoma and clinical follow-up was recommended.

All 12 patients with prolactinoma had repeated MRI and a scheduled follow-up after closure of this study. At the most recent MRI, three patients no longer had visible prolactinoma after treatment with dopamine agonists (Fig. 1). In one girl with a prolactinoma, there was initially a decrease in size from 6 to 3 mm. Later, rising prolactin levels were detected due to medication side effects. A new MRI showed an increase to the original size. One patient with a prolactinoma was operated due to problems tolerating the pharmacological treatment.

### Review of the lesion size at first and most recent MRI by an experienced neuroradiologist

**Fig. 2:**
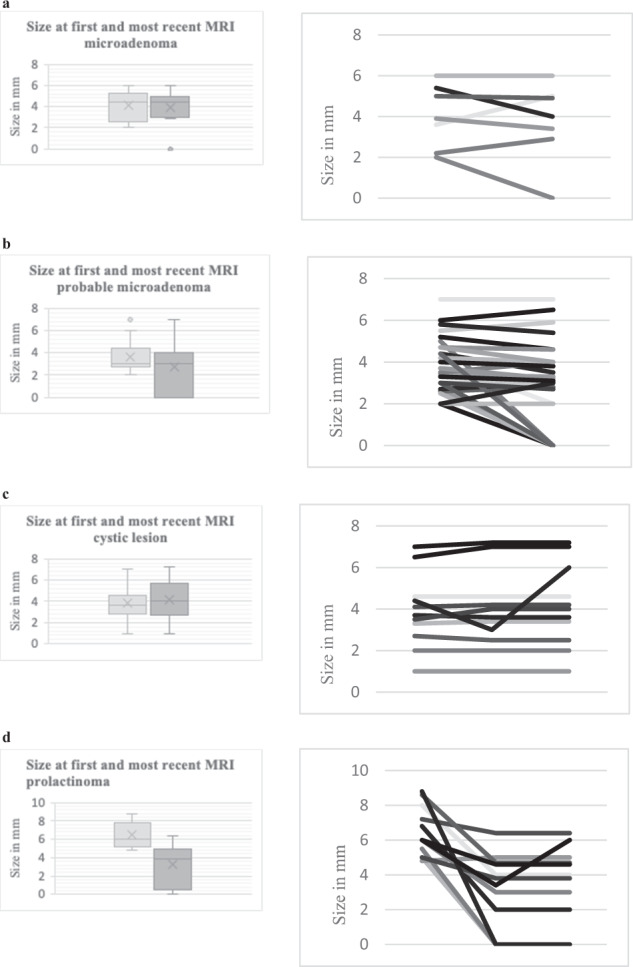
Lesion size at initial and most recent MRI as measured by an experienced neuroradiologist for (**a**) Non-functioning pituitary microadenoma (*n* = 8), (**b**) Probable microadenoma (*n* = 35), (**c**) Cystic lesion (*n* = 12), (**d**) Prolactinoma (*n* = 12). Median, range, and individual size

Of the 55 patients with repeated examinations, 37 patients were scanned only on 1.5 T and eight patients only on a 3 T MRI scanner, both at first and most recent examination. In 10 patients both field strengths were used. The size of the lesions at first and most recent MRI is presented for each group (microadenoma, probable microadenoma, cystic lesion, and prolactinoma) in Fig. 2a–d. Size was defined as largest diameter in any projection – i.e., height, width, or depth. If the lesion was not visible, the size was marked as zero.

In eight cases, the lesion was assessed primarily as a microadenoma (Fig. 2a), median size at initial MRI was 4.5 mm (range 2–6 mm), and median size at the latest MRI was 4.5 mm (range 0–6 mm). None of the eight lesions increased significantly when comparing first and last follow-up.

In 35 patients, the lesion was initially assessed as probable microadenoma (Fig. 2b). The median size for probable microadenoma at the first MRI was 3.0 mm (range 2–7 mm), and median size at the latest MRI was 3.0 mm (range 0–7 mm). In nine cases (26%), the presence of microadenoma could not be confirmed at the final MRI. After re-evaluation, none of the 35 lesions increased significantly when comparing first and last follow-up, and 10 (29%) decreased by ≥2 mm.

In 12 patients, the lesion had mainly cystic characteristics (Fig. 2c). At the initial MRI, the median size was 3.6 mm (range 1–7 mm), and the median size at the most recent MRI was 4.0 mm (range 1–7.2 mm). In all cases, a lesion in the pituitary was visible in the final scan. After re-evaluation, 11 of the 12 cystic lesions did not increase significantly when comparing the first and last examination, and none decreased significantly in size. One increased in size, as described earlier.

Of the 12 patients with a prolactinoma, the median size at initial MRI was 6.0 mm (range 4.8–8.8 mm), and the median size at the latest MRI was 3.9 mm (range 0–6.4 mm) (Fig. 2d). Three (25%) could not be detected after treatment, and seven (58%) decreased by ≥ 2 mm from the first to the last scan. In one patient, a transient increase was noted, as described earlier.

Among the children, 19 patients underwent only one MRI (Fig. 1), and 10 of these were assessed as a probable microadenoma with a median size of 2.2 mm (range 1.5–4). The remaining nine were initially assessed as cystic lesions with a median size of 2.0 mm (range 0–2.6). Two of the lesions reported as a cystic lesion in the primary report could not be visualized.

### MRI re-evaluation of measurement accuracy of lesion size by two neuroradiologists

In our study, 68.4% of the scans were performed with a 1.5 T scanner and 31.6% with a 3 T scanner. Of the 269 MRI examinations the reviewers could agree that no lesion could be identified in 38 MRI scans (14%). Of the remaining 231 examinations, the reviewers reported 180 (78%) lesions in the same quarter; that is, for 51 (22%) of the MRIs, the reviewers could not agree on lesion location. At validation of the accuracy of the MRI lesion size measurement, the median of all measurements in all three dimensions and the differences in measurements between the reviewers were calculated. All examinations where both reviewers reached an agreement concerning lesion location were identified and presented as a proportion of all reviewed examinations. The median height, width, and depth of the lesion measured by both reviewers is shown in Table 4. Not all lesions could be visualized adequately in all imaging planes. Thus, some lesions were only measured in two dimensions, which explains the differences in numbers of measurements in Table 5, so volume measurements could not be obtained. In most cases, both reviewers agreed about the size of the lesions (Table 5). However, their measurements disagreed > 2 mm in 34/460 (7%) of measurements, and >3 mm in 11/460 (2%) of measurements, which both had been considered as tumour progression. The inter-observer agreement was less accurate with the high field strength 3 Tesla (3 T) (Table 6), especially for probable microadenoma (Supplemental Table 2).

**Table 4:** The median size in mm for lesion height, width, and depth measured by both viewers, interquartile range in parenthesis, *n* = 231 MRI examinations

	Viewer 1	Viewer 2
Height	4.2 (3.0–5.7)	4.3 (3.1–5.3)
Width	4.5 (3.2–6.5)	5 (3.8–6.5)
Depth	4.4 (3.3–5.8)	4.3 (3.2–5.4)

**Table 5:** Difference in measurements of lesion size presented for all examinations where the two radiologists agreed (measured the same lesion)

	Two reviewers agreed on the same lesion n measurements	Reviewers agreed on difference > 2 mm in same lesion n (%)	Reviewers agreed on difference > 3 mm in same lesion n (%)
Height	179	8 (4.5)	3 (1.7)
Width	165	19 (11.5)	4 (2.4)
Depth	116	7 (6.0)	4 (3.5)
In total	460	34 (7.4)	11 (2.4)

Because not all lesions could be visualized adequately in all imaging planes, the number of measurements are specified for height, width, and depth. Data presented as count (percent)

**Table 6:** Inter-observer agreement of two different radiologist for MRI examinations depending on the diagnosis pituitary microadenoma, probable microadenoma, cystic lesion, prolactinoma (*n* = 269), and on the field strength (*n* = 256)

	*n* (%)	Percent agreement (95% CI)	κ (95% CI)
Microadenoma	34 (12.6)	0.853 (0.728–0.978)	0.479 (0.090–0.858)
Probable microadenoma	122 (45.4)	0.762 (0.686–0.839)	0.424 (0.246–0.603)
Cystic lesion	45 (16.7)	0.911 (0.825–0.998)	0.723 (0.459–0.987)
Prolactinoma	68 (25.3)	0.809 (0.713–0.905)	0.363 (0.078–0.646)
3T	73 (28.5)	0.740 (0.637–0.843)	0.258 (0.000–0.516)
1.5T	183 (71.5)	0.825 (0.770–0.881)	0.507 (0.358–0.656)

In 46 of 86 patients, both reviewers and the primary report noticed the lesion within the same location in the pituitary in all examinations in the same patient. Thus, in 40 patients (46.5%) there was disagreement between the reviewers themselves and/or the primary report. In the two cases with lesion enlargement, both reviewers agreed with the primary report.

## Discussion

In this retrospective study, we observed the growth potential of distinct or probable pituitary non-functioning microadenoma and cystic lesions. We also tested the measurement accuracy of the pituitary lesions, which has not been performed in previous studies, and therefore a few children with a prolactinoma were included. Among 55 children with at least one follow-up MRI, we found that non-functioning microadenoma and cysts showed small size variations, often below the spatial resolution of the scanners. In one case, there was a small increase in size over five years. In no case did the MRI surveillance finding change clinical management.

Data revealing the natural history of pituitary microadenoma/incidentaloma are still scarce, but the risk of pituitary apoplexy, visual field deficits, and endocrine dysfunction seems low in small lesions [17]. In adults, there are reports of some long-term growth of non-functioning pituitary microadenomas, although their growth seems to be slow and continues over several years. In a review [18] of eleven studies of 166 adult patients (>16 years old), 10% had enlarged lesions, 17% had decreased lesions, and 83% had stable lesions [16]. Microadenomas seldom showed progression to visual disturbances [10].

There are only four published reports that include follow-up MRI of small non-functioning pituitary lesions in children [6, 14–16], all with limited follow-up time. Repeated MRI, in these four reports, was performed in a total of 145 children. Progress was noticed in one child, but the lesion was considered stable and did not develop into a macroadenoma. These findings are in line with our results, where in one child there was an increase in size by 2 mm over five years. The radiological diagnosis of microadenoma or suspected microadenoma could possibly encompass lesions that are not microadenomas or are simply artefacts, and this could partially explain the low percentage of progressive lesions. The clinical course was the most important factor for further treatment, not the result of the MRI follow-up. Our study used an experienced neuroradiologist to re-evaluate lesion size, whereas three of the four previous studies relied on data collected in the medical records [14–16], risking considerable bias. Only in one of the previous studies in children all images were re-evaluated [6].

To our knowledge validation of the MRI accuracy, regardless of field strength (1.5 or 3 T), in detection of microadenoma has not been studied previously. In the assessment of randomized, individual MRI scans in our study, there was disagreement both between the two reviewers and between the two reviewers and the original report regarding lesion size and location. In many MRI exams (22%), the radiologists could not even agree on the location of the lesion. We found that both reviewers agreed on size in most cases, but in 7% of the measurements, they disagreed to an extent that would have been considered tumour progression. The MRI technique has obvious limitations in measurement accuracy for small pituitary lesions. Three out of the four previous studies assumed that the original radiological assessment was accurate, although the size and changes in size of many lesions are close to the spatial resolution of MRI scans. One can question the accuracy of such measurements, especially when comparing consecutive measurements where we have the bias of knowing a lesion’s location and size from a previous report. Indeed, in the second part of our study we have shown that the randomized assessment of a single scan by two neuroradiologists may vary considerably. The inter-observer agreement was less accurate with the high field strength (3 T), possibly because 3 T scanners, even though they have better spatial resolution, are more prone to artefacts caused by motion, vascular flow, and susceptibility [9].

Unnecessary MRI examinations may not add any decisive clinical information. MRI with contrast enhancement carries a risk for gadolinium brain depositions with a potential, albeit unknown, of neurotoxicity [19]. Another potential risk is the need for repeated sedation or intubation/sedation in small children. A repeated MRI should only be used on patients who might have an increase in lesion size that could indicate medical treatment or surgery – i.e., if there is a risk of visual and hormonal deficits, not just changes in size, which could be an artefact. It should also be noted that the reason for performing a MRI in many cases was clinical symptoms such as growth retardation, precocious puberty, or suspected pituitary dysfunction. In these children, clinical follow-up should be continued irrespective of the MRI finding as clinical follow-up would likely note a new symptom.

To our knowledge, there are no available consensus guidelines for children. The Endocrine Society 2011 formed guidelines for clinical and radiological follow-up for pituitary incidentaloma in adults [20]. The recommendation for a microadenoma is biochemical testing for hormone hypersecretion and follow-up with MRI after one year. MRI is then recommended every one to two years for three years and after that progressively less frequently [20]. However, Galland et al., in a French consensus document, recommends no surveillance of non-functioning microadenoma < 5 mm and for lesions ≥ 5 mm in adults a follow-up MRI after six months and then after two years. If no progression in size is seen after two years, no further MRI is needed [13].

A limitation of our study is its retrospective design, limited follow-up time, and sample size. On the other hand, strengths are the re-evaluation of lesion size and the assessment of detection and measurement accuracy of MRI as a method in this patient cohort.

In conclusion, the probability of progression for a small non-functioning pituitary microadenoma or cystic lesion in the short term is very small. The measurements that these assessments are based on are fraught with methodological limitations, as described above. Current knowledge suggests that the risk of progression of size that causes endocrine dysfunction is low in small lesions. Until there are more studies about the natural cause and risk of long-term growth of a microadenoma, we suggest the following protocols: lesions <4 mm only clinical follow-up, lesions 4–6 mm for MRI after 2 years and ≥7 mm MRI after 1 and 3 years, with clinical follow-up in between. The follow-up MRI can be performed without gadolinium as significant change in size and shape can be visualized without contrast enhancement, and cystic lesions are also well visualized this way. If no progression is observed, further MRI examinations should only be performed if there is clinical evidence of new symptoms or hormonal disturbances.

### Supplementary information

Supplementary Table 1

Supplementary Table 2

## Abbreviations

PA: probable microadenoma
CL: cystic lesion
MRI: magnetic resonance imaging
T: Tesla
