# Correction: Non-functioning pituitary microadenoma in children and adolescents: Is follow-up with diagnostic imaging necessary?

**DOI:** 10.1007/s12020-023-03541-1

**Published:** 2023-11-14

**Authors:** Camilla Borghammar, Ashkan Tamaddon, Eva-Marie Erfurth, Pia C. Sundgren, Peter Siesjö, Maria Elfving, Margareta Nilsson

**Affiliations:** 1 Skåne University Hospital, Department of Clinical Sciences, Pediatrics, Pediatric Endocrinology, Lund University, Lund, Sweden; 2 Skåne University Hospital, Department of Clinical Sciences, Medical Imaging and Physiology, Lund University, Lund, Sweden; 3 Skåne University Hospital, Department of Clinical Sciences, Endocrinology, Lund University, Lund, Sweden; 4 Department of Clinical Sciences, Radiology, Lund University, Lund, Sweden; 5 Lund Bioimaging Center, Lund University, Lund, Sweden; 6 Skåne University Hospital, Department of Clinical Sciences, Neurosurgery, Lund University, Lund, Sweden

Correction to: Endocrine

10.1007/s12020-022-03212-7 published online 17 October 2022

In the original publication of the article, the following errors has been occurred.

Introduction:

1. In children pituitary adenomas account for less than 3% of all intracranial tumours [1, 3].
2. However, a recent study found that in children below the age of 19 pituitary tumours made up 19.7% of all brain tumours and 77.9% of these were adenomas [4].

Discussion:

In a review [18] of eleven studies of 166 adult patients (>16 years old), 10% had enlarged lesions, 7% had decreased lesions, and 83% had stable lesions [16].

This has been corrected in this paper.

