# Supplementary material for: Non-functioning pituitary microadenoma in children and adolescents: Is follow-up with diagnostic imaging necessary?
Source: Endocrine. 2022 Oct 17;79(1):152–60. doi: 10.1007/s12020-022-03212-7 (PMC9813011; doi:10.1007/s12020-022-03212-7)
Supplement: Supplementary file 1 — Supplementary Table 1 [file 12020_2022_3212_MOESM1_ESM.docx]

**Supplementary Information**

**Non-functioning pituitary microadenoma in children and adolescents: Is follow-up with diagnostic imaging necessary?**

Endocrine, International Journal of Basic and Clinical Endocrinology

Camilla Borghammar^1^ MD, Ashkan Tamaddon MD, Eva-Marie Erfurth MD PhD, Pia C Sundgren MD PhD, Peter Siesjö MD PhD, Maria Elfving MD PhD, Margareta Nilsson MD PhD

^1^ Lund University, Skåne University Hospital, Institution of Clinical Sciences, Department of Pediatrics, Pediatric Endocrinology, Lund, Sweden

Corresponding author, E-mail: camilla.borghammar@med.lu.se

Supplemental Table 1. Overview of studies on nonfunctional pituitary lesions in children.

| Author,  year | Number of patients  Summary | F/M | Pituitary micro-adenoma  1/≥2 MRI | Cystic lesion  1/≥2 MRI | Follow-up time | Number of MRI | Enlargement of pituitary lesions | MRI accuracy | Recommendation/Conclusion |
| --- | --- | --- | --- | --- | --- | --- | --- | --- | --- |
| Derrick et al.,  2018 | MRI in 346 children with short stature (SS) and 215 children with precocious puberty (PP). In 105, a microadenoma was found; 54 had repeated MRI. | 20/44 (SS)  41/8 (PP) | 105/54 | 0/0 | Mean 1.75 yrs (SS)  Mean 3.57 yrs (PP) | 54 (51%) had at least one follow-up MRI | None | Not evaluated | Pituitary microadenoma is a common finding in MRI in children with SS and PP. They do not seem to be clinically significant; the majority resolved over time. Are the MRI protocols too sensitive? |
| Pedicelli et al.,  2014 | MRI in 182 girls with idiopathic central precocious puberty (CPP): 19 had a mild abnormality of the hypothalamic-pituitary region and 6 had a hamartoma. Of all the girls, 28 had repeated MRI. | 182/0 | 5/NS | 3/NS | Maximum: 9 yrs | 28 had at least one follow-up MRI (1–5) | None | Not evaluated | MRI finding suggest that microadenoma in girls with central precocious puberty has an uncertain pathophysiological and clinical significance. |
| Souterio et al.,  2019 | MRI in 41 children with pituitary incidentaloma (wide definition of pituitary incidentaloma*). Of all exams, 70.7% MRI and 29.3% CT-scan. 23 children had repeated scans, all MRI. 90.2% had no symptoms of hypopituitarism or hormonal hypersecretion. | 26/15 | 5/NS | 5/NS | Median 24.6 months | 23 (56%) had at least one follow-up MRI | None | Not evaluated | Lower risk for of progression of pituitary incidentalomas in children than in adults. |
| Thaker et al.,  2019 | 78 children with pituitary cyst, microadenoma, or possible microadenoma. Results reported for 44 children with microadenoma; 40 underwent repeated MRI. | 36/8 | 44/40 | (34/NS) | First MRI – last visit: mean 4.5 yrs (+/- 2.6) | 40 had at least on follow-up MRI, median (range), 2 (1–10) | 1/40. One microadenoma increased 4 mm, but remained < 10 mm after 6 years. | Not evaluated | Cystic lesions, possible microadenoma, and microadenoma in children are benign. In absence of endocrine or visual disturbances, repeated MRI is not needed. If repeated MRI is performed, it should be done after no less than one year. |
| Our study | 74 children with microadenoma, probable microadenoma (PA), or cystic lesion (CL); 55 with repeated MRI. | 43/31 | 53/43 (Micro-adenoma and PA) | 21/12 | Median (range), 37 months (4–189) | Median (range), 3 (2–7) MRI | 1/55. Enlargement of one cystic lesion from 4 to 6 mm along with clinical signs of progression and hormonal deficits. | Evaluated | The probability of progression for a small non-functioning pituitary microadenoma or cystic lesion is very small. Suggested follow-up: for lesions < 4 mm, no further MRI; for lesions 4–6 mm, MRI after 24 months, and for lesions ≥7mm MRI after 1 and 3 years. |

*Wide definition of pituitary incidentaloma including pituitary hypertrophy, arachnoid cysts, adenoma, Rathke’s cleft cyst, thickened pituitary stalk, hamartoma, craniopharyngioma, etc.

**Abbreviations**: F, female; M, male; NA, not applicable; NS, not specified; yrs, years; PA, probable microadenoma; CL, cystic lesion; MRI, magnetic resonance imaging; CT, computerized tomography; SS, short stature; PP, precocious puberty; CPP central precocious puberty
