# Supplementary material for: Non-functioning pituitary microadenoma in children and adolescents: Is follow-up with diagnostic imaging necessary?
Source: Endocrine. 2022 Oct 17;79(1):152–60. doi: 10.1007/s12020-022-03212-7 (PMC9813011; doi:10.1007/s12020-022-03212-7)
Supplement: Supplementary file 2 — Supplementary Table 2 [file 12020_2022_3212_MOESM2_ESM.docx]

**Supplementary Information**

**Non-functioning pituitary microadenoma in children and adolescents: Is follow-up with diagnostic imaging necessary?**

Endocrine, International Journal of Basic and Clinical Endocrinology

Camilla Borghammar^1^ MD, Ashkan Tamaddon MD, Eva-Marie Erfurth MD PhD, Pia C Sundgren MD PhD, Peter Siesjö MD PhD, Maria Elfving MD PhD, Margareta Nilsson MD PhD

^1^ Lund University, Skåne University Hospital, Institution of Clinical Sciences, Department of Pediatrics, Pediatric Endocrinology, Lund, Sweden

Corresponding author, E-mail: camilla.borghammar@med.lu.se

Supplemental Table 2. Inter-observer agreement of two different viewers depending on the diagnosis and field strength of MRI examination, n = 256.

| **Diagnosis/Field strength** | **n (%)** | **Percent agreement (95% CI)** | **κ (95% CI)** |
| --- | --- | --- | --- |
| **Microadenoma, n = 30** |  |  |  |
| 3T | 5 (16.7) | 0.800 (0.245–1.000) | 0.546 (-0.579–1.000) |
| 1.5T | 25 (83.3) | 0.840 (0.687–0.994) | 0.000 (-0.000–0.000)⁕ |
| **Probable microadenoma, n = 118** |  |  |  |
| 3T | 38 (32.2) | 0.632 (0.471–0.792) | 0.104 (-0.240–0.448) |
| 1.5T | 80 (67.8) | 0.813 (0.725–0.900) | 0.537 (0.324–0.747) |
| **Cystic lesion, n = 42** |  |  |  |
| 3T | 13 (31.0) | 0.923 (0.756–1.000) | 0.629 (-0.123–1.000) |
| 1.5T | 29 (69.0) | 0.897 (0.779–1.000) | 0.731 (0.428–1.000) |
| **Prolactinoma, n = 66** |  |  |  |
| 3T | 17 (25.8) | 0.824 (0.622–1.000) | 0.338 (-0.231–0.906) |
| 1.5T | 49 (74.2) | 0.796 (0.679–0.913) | 0.373 (0.048–0.698) |

⁕ Since one rater consistently did not vary his/her ratings, kappa (κ) is zero by definition.
